# Postural Control Adaptations in Trampoline Athletes of Different Competitive Levels: Insights from COP Linear and Nonlinear Measures

**DOI:** 10.3390/e27121181

**Published:** 2025-11-21

**Authors:** Mengzi Sun, Fangtong Zhang, Xinglong Zhou, Feng Qu, Wenhui Mao, Li Li

**Affiliations:** 1 School of Sports Science and Physical Education, Nanjing Normal University, Nanjing 210023, China; 12199@njnu.edu.cn (M.S.);; 2 Biomechanics Laboratory, Beijing Sport University, Beijing 100084, China; 3 Department of Health Sciences and Kinesiology, Georgia Southern University, Statesboro, GA 30460, USA

**Keywords:** trampoline gymnastics, postural control, center of pressure (COP), sample entropy (SampEn), training adaptation

## Abstract

Balance is a fundamental quality for trampoline athletes, the basis for completing complex skills. We aimed to compare balance control strategies between elite trampolinists (ETs) and sub-elite trampolinists (Sub-ET) by integrating linear and nonlinear center of pressure (COP) measures across stable and unstable surfaces. Twenty-four male athletes (12 ET, 12 Sub-ET) participated. Each participant performed 15-s static standing trials with eyes closed on a firm surface (FI) and a foam surface (FO). COP parameters were extracted, including ellipse area, sway velocity, sway range, and sample entropy (SampEn) in the medio-lateral (ML) and antero-posterior (AP) directions. Repeated-measures ANOVA was applied to examine the effects of group and surface condition. Linear analyses indicated that ET athletes exhibited greater sway amplitudes and faster velocities than Sub-ET athletes, with both groups showing larger sway on FO compared with FI. Nonlinear analyses revealed that ET athletes demonstrated lower SampEn, suggesting more structured and automatized control strategies. ET athletes maintained consistent entropy across both conditions, reflecting stronger adaptability to unstable surfaces. These results emphasize the importance of combining linear and nonlinear measures in balance assessment and suggest that incorporating unstable or trampoline-like surfaces into training may enhance adaptability, improve performance, and reduce injury risk.

## 1. Introduction

Balance ability refers to an individual’s capacity to maintain postural stability under both static and dynamic conditions [1]. For trampoline athletes, balance represents a fundamental physical quality, as it is essential for executing complex somersaults and twists, maintaining body stability in flight, and ensuring safe and accurate landings. Consequently, balance ability is closely linked to both performance outcomes and injury risk [2].

Investigations of balance control in athletes have typically employed both linear and nonlinear analytical approaches, as each captures different aspects of postural regulation [3]. Linear center of pressure (COP) parameters, such as area, velocity, and range, are widely used to quantify postural variability, with smaller values generally interpreted as reflecting stronger balance and reduced sway variability [4]. In contrast, nonlinear measures describe the temporal dynamics of CoP displacement, reflecting how motor behavior changes over time. These measures provide valuable information about the regularity, adaptability, stability, and complexity of postural control [5]. Sample entropy (SampEn) provides a nonlinear measure that reflects the complexity of COP time series and the refinement of neural control. Lower SampEn values indicate greater regularity and reduced complexity [6].

Previous studies have shown that athletes often display smaller COP ranges but higher sway velocities than non-athletes, suggesting that enhanced postural efficiency may enable quicker compensatory responses and greater precision in motor timing [7,8]. Postural control is also shaped by sport-specific demands, habitual training patterns, and the surface [9]. Federolf and coworkers [10] reported no differences between athletes and non-athletes during quiet standing; however, in sport-specific postures such as the shooting stance, athletes demonstrated lower SampEn, which reflected a more refined control strategy rather than impaired balance. Conversely, Akbaş and colleagues [11] observed higher entropy values in ju-jitsu athletes compared with controls when adopting a combat stance, despite no differences during quiet standing. These contrasting findings underscore that entropy-based measures may vary across sports and task contexts, highlighting the importance of examining balance within discipline-specific conditions.

Differences across competitive levels have also been reported. Linear COP indices often lack sensitivity for distinguishing balance ability among athletes, whereas nonlinear measures such as SampEn appear to offer greater discriminatory power [3]. For example, elite athletes have been found to exhibit smaller and more stable COP areas, accompanied by higher entropy values and more complex control patterns, while sub-elite athletes show greater sway variability and reduced entropy [12]. These findings suggest that combining linear and nonlinear analyses provides a more comprehensive assessment of balance ability.

Previous research has extensively utilized linear COP indices (e.g., sway velocity, path length, and area) to compare the static balance ability of athletes in skill-based sports such as gymnastics and soccer [13,14,15]. These studies have substantially advanced our understanding of how expertise modulates sway magnitude and variability. However, despite the importance of dynamic postural control on highly unstable surfaces, research specifically targeting trampoline gymnastics remains limited. More critically, most prior investigations have primarily focused on the quantity of sway (i.e., how much the body moves), while overlooking the quality of sway dynamics—namely, the temporal structure that reflects neuromuscular adaptability and control sophistication.

From the perspective of complexity science, SampEn measures how predictable a signal is by examining how often similar patterns remain similar over time, which reflects the signal’s regularity at a single time scale. Multiscale Entropy (MSE) is based on it by calculating SampEn across several time scales, allowing researchers to see how signal complexity changes from short to long-term patterns [16,17]. In short, SampEn focuses on local regularity, whereas MSE provides a broader view of how complex and adaptive the control system is across multiple time scales [18]. Methods such as SampEn offer valuable insights into the adaptive nature of postural regulation [19,20]. Higher complexity (greater entropy) typically represents a more flexible and resilient neuromuscular system capable of responding effectively to perturbations, whereas lower complexity may indicate rigid or overly constrained control, often associated with either functional degradation or the demands of highly constrained tasks [21,22]. Therefore, a sole reliance on linear metrics may obscure important qualitative differences in postural control strategies between athletes of differing expertise levels.

Building on this theoretical foundation, the present study integrates traditional linear COP indices with nonlinear complexity metrics to examine balance control among elite (ET) and sub-elite (Sub-ET) trampoline athletes under firm and foam surface conditions. Rather than merely contrasting “how much” postural sway differs, this approach seeks to reveal “how” balance control strategies differ—specifically, how the neuromuscular system organizes and refines postural adjustments in response to varying surface stability.

Thus, the present study examined the balance control characteristics of elite (ET) and sub-elite (Sub-ET) trampoline athletes using linear and nonlinear COP measures under firm and foam surface conditions. We hypothesized that (1) ET would demonstrate greater variability but more regular COP structures than Sub-ET athletes, and (2) under the firm surface condition, the balance control of ET and Sub-ET athletes would appear comparable, whereas under the foam surface condition, ET athletes were expected to demonstrate more stable and efficient control strategies.

Postural control assessment leverages various technologies, with inertial measurement units (IMUs) and force platforms being widely adopted. While IMUs offer portability for field studies, force platforms provide high-precision, direct measurement of the center of pressure (COP) trajectory under standardized laboratory conditions [23,24,25]. For the specific purpose of this study—to conduct a detailed analysis of both linear and non-linear dynamics from the COP signal during quiet standing—a force platform was employed to ensure the required data accuracy.

## 2. Materials and Methods

### 2.1. Participants

The study was reviewed and approved by the Sports Science Experiment Ethics Committee of Beijing Sport University (Approval No. 2024090H). A total of 24 male trampoline athletes from the Fujian and Shanxi provincial teams participated in the experiment. To be included, athletes were required to be professional members of provincial teams with access to national training resources and facilities. They were also required to have achieved at least the National Master level (elite trampolinists, ET) or National First-Class level (sub-elite trampolinists, Sub-ET), with demonstrated records of competitive success at the national or international level.

All participants were expected to have maintained relatively continuous trampoline training for at least three years. The athletes trained six days per week (approximately 6 h per day), including four days of sport-specific trampoline training, one day of physical conditioning, and one day dedicated to coordination training. During the coordination training sessions, athletes performed balance exercises on various planes to enhance postural control and stability. Participants were excluded if they had chronic conditions affecting postural control or if they were receiving treatment for, or had not fully recovered from, sports-related injuries within the previous six months.

The coaches signed participation in the study for the younger athletes. And the younger ones received informed consent from parents through their coaches. Based on the competitive level, participants were divided into two groups. The ET group consisted of 12 male athletes with a mean age of 21.6 ± 3.5 years, height of 170.5 ± 4.9 cm, body mass of 60.25 ± 6.34 kg, and an average of 13.8 ± 4.9 years of training experience. The Sub-ET group also included 12 male athletes, who were younger with a mean age of 13.5 ± 1.2 years, height of 151.4 ± 8.4 cm, body mass of 39.96 ± 6.21 kg, and an average training history of 6.8 ± 1.5 years.

### 2.2. Procedures

The tests were conducted before the athletes’ regular training sessions, ensuring that they were in a non-fatigued state. All tests were performed at the training gym under a controlled environmental temperature of 24 °C. All athletes performed their routine pre-training warm-up, which consisted of 10 min of jogging. Ground reaction forces were recorded using a three-dimensional force platform (Kistler 9281CA, Kistler Instrumented AG, Winterthur, Switzerland). The platform surface was used as the firm (FI) surface condition, while a TPE square balance pad (Jointfit, Suzhou, China) placed on top of the platform served as the foam (FO) surface condition. In both conditions, participants maintained a static upright stance with feet together and eyes closed for 15 s [26]. During testing, participants kept their arms relaxed at their sides, avoided unnecessary movements, and refrained from using visual feedback. The COP trajectories were recorded in both antero-posterior (AP) and medio-lateral (ML) directions. The sampling frequency of the acquisition was 1000 Hz. Each condition was tested three times, with sufficient rest between trials.

Although dynamic balance plays a critical role in trampoline performance, this study intentionally focused on static balance assessment for two key reasons. First, static postural control forms the fundamental basis of dynamic stability. A neuromuscular system that cannot maintain efficient control under static conditions is unlikely to perform effectively during complex, rapidly changing movements. Therefore, examining static balance mechanisms offers essential insight into the underlying neural and biomechanical control processes that support dynamic performance [27,28,29]. Second, this study represents the initial stage of a progressive research framework. Establishing a static baseline allows for future comparisons with dynamic balance evaluations—such as landing stability or aerial posture control—providing a valid reference point for understanding how balance control adapts with increased task complexity [19].

### 2.3. Data Processing

All data analysis was conducted in MATLAB R2015a (The MathWorks Inc., Natick, MA, USA). Raw AP and ML COP time-series were filtered using a fourth-order, zero-phase lag Butterworth low-pass digital filter with a 50-Hz cut-off frequency [30]. The filtered AP and ML data were subsequently processed to extract both linear and nonlinear measures of postural control. The linear parameters included the COP ellipse area, total velocity, ML velocity, AP velocity, ML range, and AP range. In addition, nonlinear characteristics of COP dynamics were assessed by calculating SampEn, which provided insights into the regularity and complexity of postural sway patterns [31]. The SampEn of AP and ML was calculated, where *m* = 2, *r* = 0.2, and *n* = 15,000 were used [32].

### 2.4. Statistics

All data were processed using SPSS 26.0 (IBM Inc., Chicago, IL, USA). The normality was calculated using the Shapiro–Wilk method. Outliers were identified and removed prior to analysis. A two-factor (surface condition × group) mixed-design ANOVA was used to examine the differences in each variable. Statistical significance was set at α = 0.05. Effect sizes were reported using partial eta squared (*η*^2^), with thresholds of 0.01, 0.06, and 0.14 representing small, medium, and large effects, respectively [33]. Values are reported as Mean ± SD.

## 3. Results

**Figure 1:**
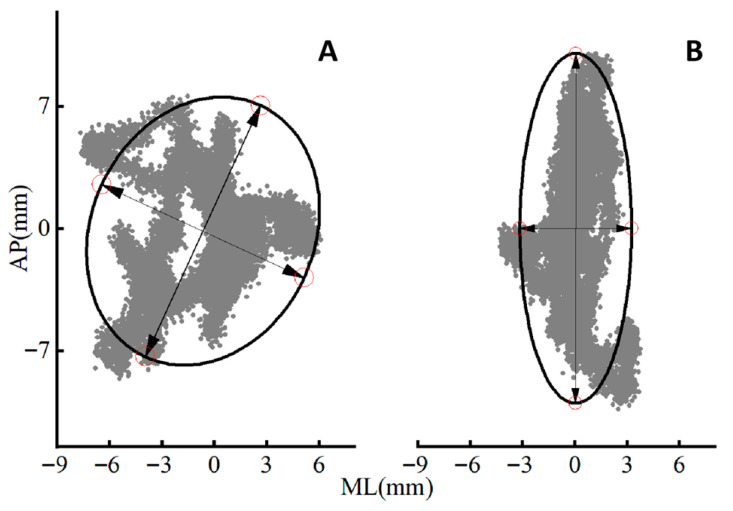
Exemplary COP trajectory and ellipse area among Sub-ET (**A**) and ET (**B**) in the graphs.

The repeated measures ANOVA revealed significant differences in nonlinear and linear COP parameters between groups and across surface conditions (see Table 1 for more details). The exemplary COP trajectory and ellipse area among Sub-ET and ET groups are shown in Figure 1.

**Figure 2:**
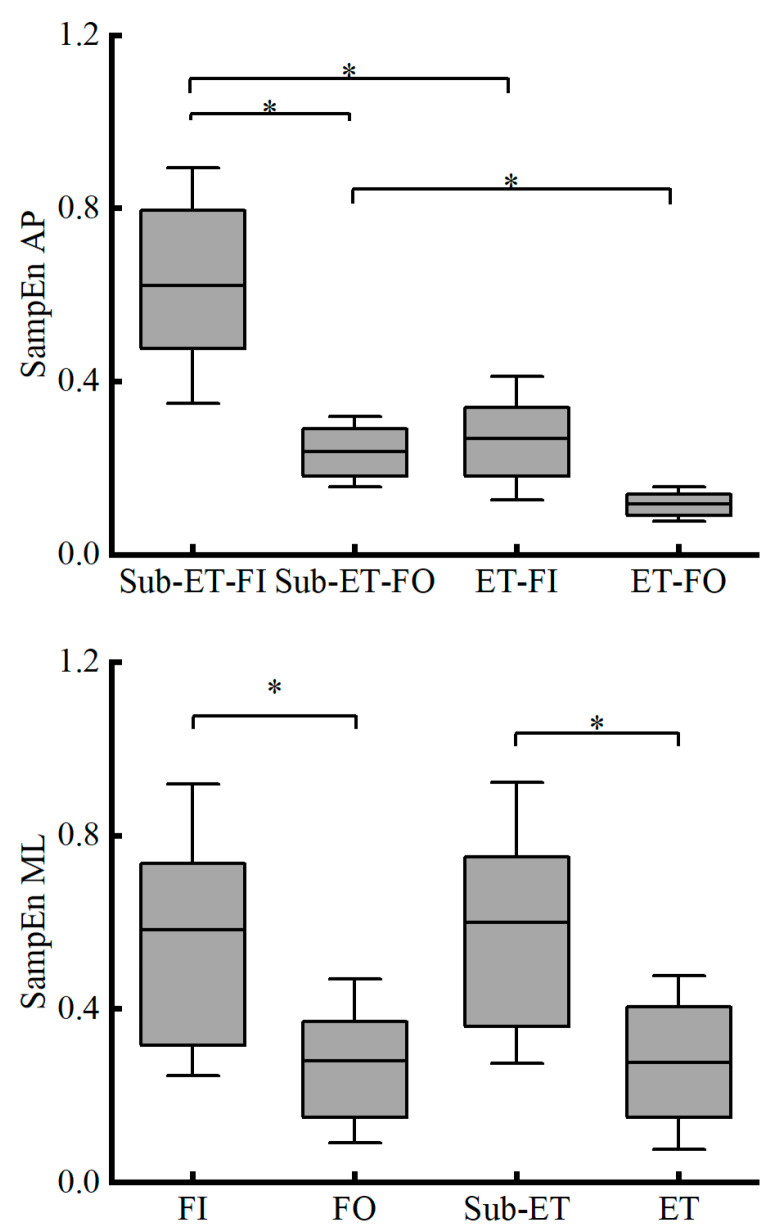
SampEn-AP (Mean ± SD) (**up panel**) and SampEn-ML (Mean ± SD) (**down panel**) directions for two groups (ET and Sub-ET) under two surface conditions (FI and FO). * Indicates a significant difference. FI refers to firm surface; FO refers to foam surface; Sub-ET refers to sub-elite trampolinists; ET refers to elite trampolinists.

For medio-lateral sample entropy (SampEn-ML), no significant interaction was detected between athlete level and surface condition (F_1,21_ = 1.848, *p* = 0.188, partial *η*^2^ = 0.081). Nevertheless, the ET group demonstrated significantly lower SampEn values than the Sub-ET group (*p* = 0.001, partial *η*^2^ = 0.419). SampEn-ML was also reduced considerably under FO relative to FI (*p* < 0.001, partial *η*^2^ = 0.650) (see Figure 2 for more details).

A significant interaction effect was found in the antero-posterior sample entropy (SampEn-AP) (F_1,21_ = 6.304, *p* = 0.010, partial *η*^2^ = 0.277). Post hoc analyses indicated that the Sub-ET group exhibited significantly lower SampEn values in the FO condition compared with the FI condition (*p* < 0.001). In contrast, no significant difference was observed between FO and FI in the ET group (*p* = 0.079). Moreover, under both FI (*p* < 0.001) and FO (*p* < 0.001) conditions, the ET group consistently demonstrated significantly lower SampEn values than the Sub-ET group.

**Figure 3:**
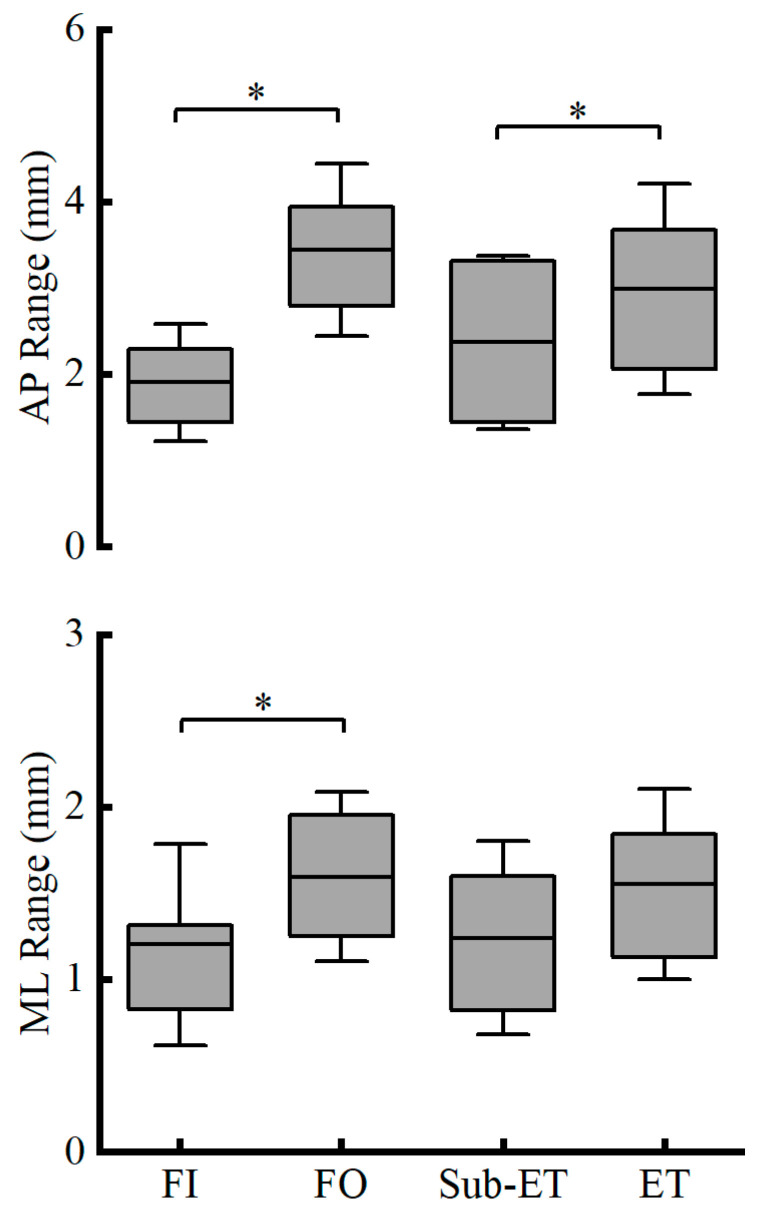
COP range in the AP (Mean ± SD) (**up panel**) and ML (Mean ± SD) (**down panel**) for two groups (ET and Sub-ET) under two surface conditions (FI and FO). * Indicates a significant difference. FI refers to firm surface; FO refers to foam surface; Sub-ET refers to sub-elite trampolinists; ET refers to elite trampolinists.

As to the COP range, no significant interaction was observed in the ML direction (F_1,20_ = 0.034, *p* = 0.856, partial *η*^2^ = 0.002), and group differences were not significant (*p* = 0.240, partial *η*^2^ = 0.068). However, values were significantly greater under FO than FI (*p* < 0.001, partial *η*^2^ = 0.660). In contrast, the AP range showed no interaction (*p* = 0.996, partial *η*^2^ < 0.001) but was significantly larger in the ET group than in the Sub-ET group (*p* = 0.007, partial *η*^2^ = 0.302), and was also significantly greater under FO relative to FI (*p* < 0.001, partial *η*^2^ = 0.650) (see Figure 3 for more details).

**Figure 4:**
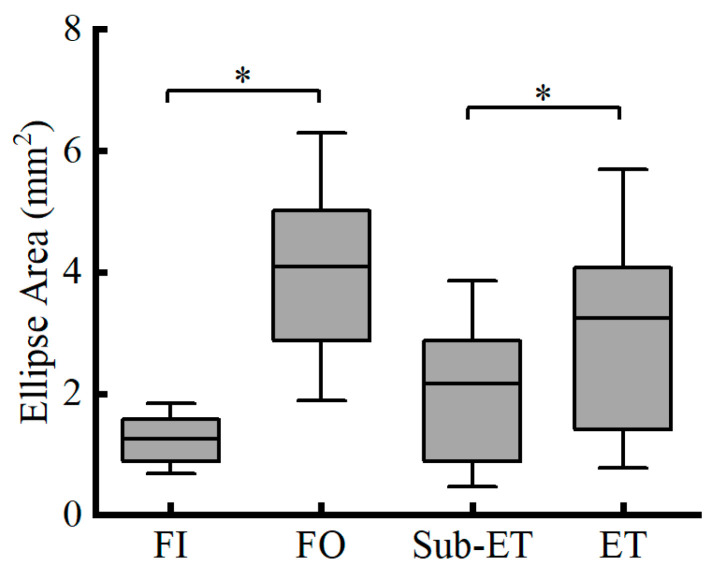
COP ellipse area (Mean ± SD) for two groups (ET and Sub-ET) under two surface conditions (FI and FO). * Indicates a significant difference. FI refers to firm surface; FO refers to foam surface; Sub-ET refers to sub-elite trampolinists; ET refers to elite trampolinists.

For the COP ellipse area, no significant interaction was observed (F_1,20_ = 2.312, *p* = 0.144, partial *η*^2^ = 0.104). Nonetheless, the ET group exhibited significantly larger ellipse areas compared with the Sub-ET group (*p* = 0.013, partial *η*^2^ = 0.271), and ellipse areas were significantly greater under FO than FI (*p* < 0.001, partial *η*^2^ = 0.610) (see Figure 4 for more details).

**Figure 5:**
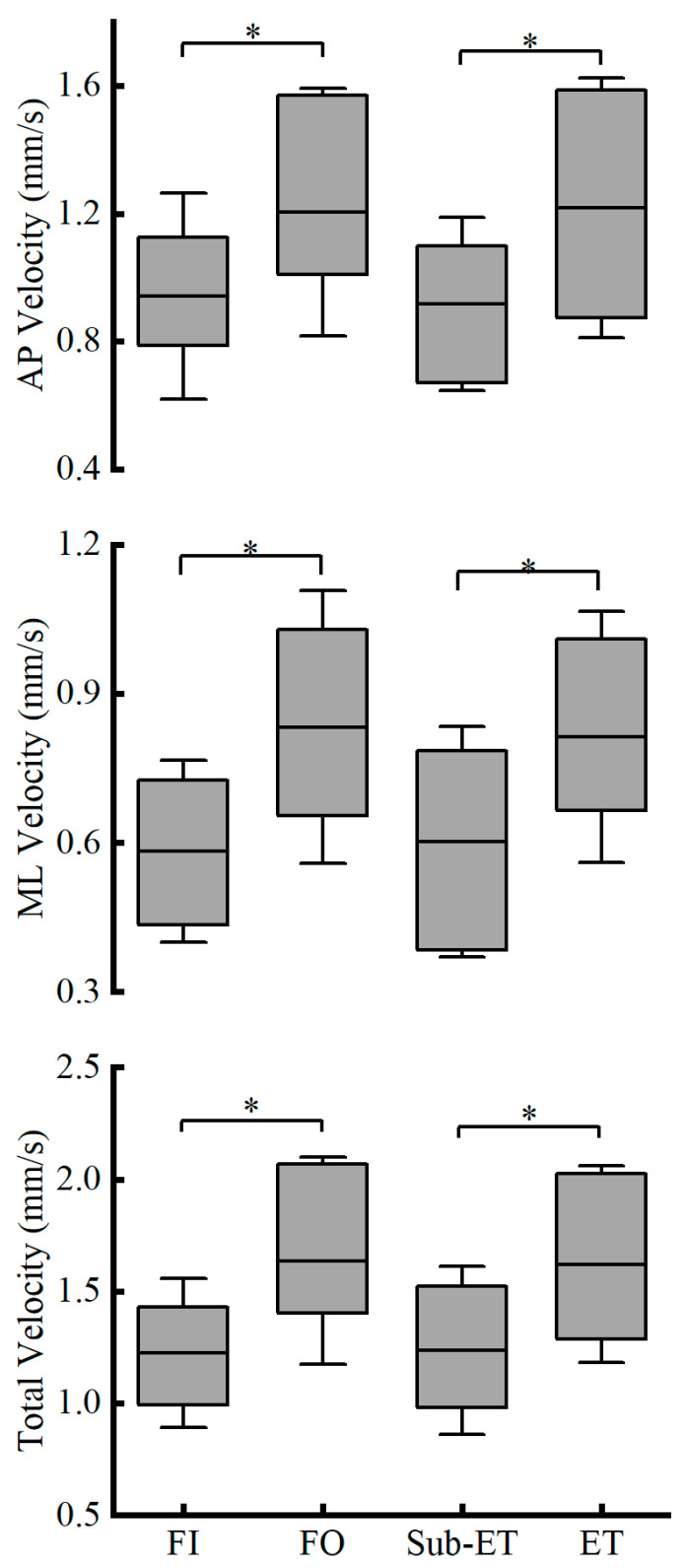
Velocity in the AP, ML, and total directions (Mean ± SD) shown from top to bottom panel under two surface conditions (FI and FO). * Indicates a significant difference. FI refers to firm surface; FO refers to foam surface; Sub-ET refers to sub-elite trampolinists; ET refers to elite trampolinists.

Analysis of COP velocity parameters revealed no significant interaction effects (all *p* > 0.05). However, the ET group consistently showed significantly higher ML velocity (*p* = 0.010, partial *η*^2^ = 0.267), AP velocity (*p* = 0.012, partial *η*^2^ = 0.255), and total velocity (*p* = 0.003, partial *η*^2^ = 0.327) compared with the Sub-ET group. Furthermore, all velocity measures were significantly greater under FO relative to FI, including medio-lateral velocity (*p* < 0.001, partial η^2^ = 0.608), antero-posterior velocity (*p* = 0.001, partial *η*^2^ = 0.395), and total velocity (*p* < 0.001, partial *η*^2^ = 0.512) (Figure 5).

## 4. Discussion

This study revealed clear differences in balance control between ET and Sub-ET athletes across linear and nonlinear indices. In summary, ET displayed greater sway amplitudes and faster velocities than Sub-ET while exhibiting more structured and predictable control patterns. By contrast, Sub-ET maintained smaller sway magnitudes but relied on more variable and less efficient regulation strategies.

It is important to first acknowledge the conventional interpretation widely reported in the literature. In clinical populations (e.g., populations with balance disorders, elderly individuals) and non-athletic groups, larger COP displacement, area, or velocity is typically associated with poorer postural stability and reduced control efficiency [5,22]. This perspective has provided a robust framework for identifying motor deficits and fall risks in pathological or untrained populations.

However, when examining high-level athletes, this interpretation may not fully apply. Elite performers operate under fundamentally different neuromechanical and perceptual conditions. In this context, we propose that greater COP variability reflects a deliberate, proactive, and exploratory sensorimotor strategy, rather than instability or poor control. The evidence from studies on postural control under threat or instability. When exposed to threatening or unstable environments, experienced individuals often exhibit larger COP variability, which represents adaptive exploratory behavior rather than instability. Such responses are thought to enhance sensory feedback and readiness to counter perturbations [34].

Similarly, trampoline performance occurs in an inherently unstable and threat-like environment, demanding constant postural adjustment. Accordingly, the larger yet well-structured COP fluctuations observed in ET athletes may reflect an optimal variability that supports flexible and efficient adaptation to instability. In contrast, the smaller sway magnitudes of Sub-ET athletes likely indicate a more constrained and conservative control mode, prioritizing short-term stability at the expense of adaptability. Elite athletes appear to tolerate and regulate larger COP variability with confidence, reflecting a system that perceives balance perturbations as less threatening, while reduced entropy, as noted in previous research [10], may represent a more efficient form of postural control achieved by minimizing unnecessary adjustments. It indicates a form of robust stability in which flexibility and precision coexist within a dynamically stable system.

Consistent with this reasoning, prior studies have reported that athletes often exhibit increased sway velocities and areas compared with non-athletes, particularly under challenging or unstable conditions [35,36,37]. Such patterns have been interpreted as reflecting more responsive and finely tuned neuromuscular control, rather than instability. Similarly, in our data, ET athletes combined higher linear indices (ellipse area, velocity, range) with lower SampEn, suggesting that their larger sway is not random but systematically organized—an efficient strategy for maintaining control within an expanded stability boundary.

The nonlinear observations complement this interpretation. The regularity and predictability of the COP time series can be assessed using entropy-based measures [16,38,39,40,41,42,43]. Lower entropy values indicate more regular and predictable patterns, whereas higher values suggest irregularity and unpredictability [31]. Lower entropy values, especially in the anterior–posterior direction, indicate more predictable and consistent COP trajectories, signifying an automatized and efficient control mode. This pattern should not be interpreted as rigidity but as evidence of economic regulation, minimizing unnecessary corrections while preserving readiness. By contrast, the higher entropy in Sub-ET athletes suggests more irregular and less automatized control, providing flexibility but at the expense of stability.

The observed postural profile in elite athletes—characterized by elevated COP velocity and range with reduced SampEn. It presents a paradox when viewed against established literature, which typically interprets larger sway as instability and higher entropy as adaptability [4,5,11]. However, these interpretations derive largely from static posture studies in clinical or non-athletic populations. For trampoline athletes, the functional goal is not to remain motionless but to generate a stable and rhythmic platform for repeated take-offs and landings. Thus, the coexistence of greater sway and lower SampEn likely reflects a practiced, efficient, and highly consistent control strategy tailored to this repetitive task. It is consistent with findings in other precision-oriented sports such as rifle shooting [10]. Therefore, we propose that in this context, the coexistence of larger sway and higher regularity does not indicate a deficit, but an optimization. The athletes confidently use a wider spatial range for balance adjustments, while their nervous system produces exceptionally consistent and economical commands to control that movement. This highlights that postural control metrics must be interpreted within the specific functional context of the task and the population. And a profile combining greater sway with higher regularity can be a trait of elite performance in sports requiring rhythmic, precise generation of force.

Both ET and Sub-ET exhibited greater sway ranges and velocities on foam compared to firm surfaces, consistent with the reduced reliability of ankle and cutaneous inputs under compliant conditions [44]. However, nonlinear measures revealed divergent strategies. For the Sub-ET group, SampEn decreased on foam, indicating a more regular, less complex, and predictable control mode [45]. Rather than representing improved stability, it reflects a reactive adjustment to environmental instability, a sign that their control system remains sensitive to surface changes and has not yet formed a stable, robust balance strategy. In contrast, ET athletes maintained similar entropy in both surface conditions, suggesting that the unstable surface did not disrupt their postural organization. This consistency in sway complexity leads to a highly automatized and resilient control system, capable of maintaining adaptive variability despite environmental perturbations. Such stability of nonlinear structure may reflect the deep, experience-based understanding of movement dynamics developed through long-term trampoline training.

Taken together, these findings emphasize the importance of integrating linear and nonlinear analyses to understand athletic balance control. Linear measures capture the magnitude and speed of sway, while nonlinear indices reveal the organization and adaptability of underlying control strategies [5,38]. ET athletes combined greater variability with more structured patterns, representing efficient, automatized regulation [10], whereas Sub-ET athletes relied on smaller sway amplitudes but less efficient strategies.

Several limitations should be noted. Firstly, the relatively small and homogeneous sample limits the generalizability of the observations, and future studies should include larger and more diverse athlete groups. Also, there is an age difference between the two groups of athletes. Their height and body mass differ as well, and these factors can influence postural control from a biomechanical perspective. This situation is common in competitive sports. Future studies should select age-matched athletes of different skill levels for research. Secondly, this study examined only static balance, whereas trampoline performance primarily depends on dynamic balance. Incorporating dynamic or perturbation-based tasks would provide more ecologically valid insights. Finally, combining COP data with electromyographic or neural recordings could yield a deeper understanding of the mechanisms underlying postural control.

In summary, the present observations contribute to a deeper understanding of balance regulation in elite athletes. Greater sway variability does not necessarily indicate poorer balance but may instead represent a structured and adaptive control strategy. By integrating linear and nonlinear COP measures, this study advances a more comprehensive framework for evaluating postural regulation. Practically, these insights highlight the need for balance training programs that go beyond simply minimizing sway, emphasizing instead adaptive control and proprioceptive exploration, particularly on unstable or trampoline-like surfaces, to enhance athletic performance and resilience.

## 5. Conclusions

This study demonstrates that elite trampoline athletes achieve balance by combining greater sway variability with highly structured and automatized control strategies, allowing them to maintain stability within wider margins of movement. Importantly, unlike sub-elite athletes who rely on less complex and more rigid control, they also sustain consistent regulatory patterns under unstable conditions. The results of the present study suggest that balance training for developing young athletes should not only aim to minimize sway but also cultivate adaptive and efficient control strategies across different surface conditions. Incorporating exercises that challenge stability on unstable or trampoline-like surfaces may enhance the adaptability and resilience of postural regulation, contributing to improved performance and reduced injury risk.

## Figures and Tables

**Table 1:** The linear and nonlinear outcomes of the mixed-design ANOVA between Sub-ET and ET groups under two surface conditions (FI and FO).

COP Parameters	Group	FI	FO	F Value	Competitive Level		Surface Condition		Competitive Level * Surface Condition	
					*p*	Partial η^2^	*p*	Partial η^2^	*p*	Partial η^2^
SampEn-ML	Sub-ET	0.73 ± 0.27	0.40 ± 0.20	F_1,21_ = 1.848	0.001 *	0.419	<0.001 *	0.650	0.188	0.081
	ET	0.38 ± 0.23	0.17 ± 0.08							
SampEn-AP	Sub-ET	0.62 ± 0.27	0.24 ± 0.08	F_1,21_ = 6.304	-	-	-	-	0.010 *	0.277
	ET	0.24 ± 0.11	0.12 ± 0.04							
ML Range (mm)	Sub-ET	0.99 ± 0.4	1.49 ± 0.59	F_1,20_ = 0.034	0.240	0.068	<0.001 *	0.660	0.856	0.002
	ET	1.17 ± 0.34	1.71 ± 0.32							
AP Range (mm)	Sub-ET	1.53 ± 0.4	3.04 ± 0.69	F_1,21_ < 0.001	0.007 *	0.302	<0.001 *	0.650	0.996	<0.001
	ET	2.24 ± 0.73	3.74 ± 1.16							
Ellipse Area (mm^2^)	Sub-ET	1.01 ± 0.52	3.3 ± 1.71	F_1,20_ = 2.312	0.013 *	0.271	<0.001 *	0.610	0.144	0.104
	ET	1.72 ± 0.86	5.72 ± 3.2							
ML Velocity (mm/s)	Sub-ET	0.49 ± 0.14	0.71 ± 0.26	F_1,22_ = 0.598	0.010 *	0.267	<0.001 *	0.608	0.448	0.026
	ET	0.67 ± 0.18	0.95 ± 0.24							
AP Velocity (mm/s)	Sub-ET	0.84 ± 0.24	1.05 ± 0.3	F_1,22_ = 0.865	0.012 *	0.255	0.001 *	0.395	0.362	0.038
	ET	1.04 ± 0.32	1.39 ± 0.34							
Total Velocity (mm/s)	Sub-ET	1.07 ± 0.26	1.4 ± 0.41	F_1,22_ = 0.929	0.003 *	0.327	<0.001 *	0.512	0.345	0.041
	ET	1.38 ± 0.33	1.87 ± 0.4							

* indicates statistically significant difference (*p* < 0.05). FI refers to firm surface; FO refers to foam surface; Sub-ET refers to sub-elite trampolinists; ET refers to elite trampolinists.

## Data Availability

Data supporting reported results can be obtained from the first author.
